# Body image, self-esteem, and sense of masculinity in patients with prostate cancer: a qualitative meta-synthesis

**DOI:** 10.1007/s11764-021-01007-9

**Published:** 2021-05-08

**Authors:** Jessica Bowie, Oliver Brunckhorst, Robert Stewart, Prokar Dasgupta, Kamran Ahmed

**Affiliations:** 1 MRC Centre for Transplantation, Guy’s Hospital Campus, King’s College London, King’s Health Partners, London, UK; 2 King’s College London Institute of Psychiatry, Psychology and Neuroscience, London, UK; 3 South London and Maudsley NHS Foundation Trust, London, UK; 4 Department of Urology, King’s College Hospital, London, UK

**Keywords:** Body image, Cancer, Masculinity, Mental health, Oncology, Prostate cancer

## Abstract

**Purpose:**

Body image, self-esteem, and masculinity are three interconnected constructs in men with prostate cancer, with profound effects on quality of life. This meta-synthesis aimed to evaluate all known qualitative studies published studying the effect of prostate cancer on these constructs.

**Methods:**

A systematic review utilising PubMed, Embase, MEDLINE, and PsycINFO databases up to May 2020 was conducted in line with PRISMA and ENTREQ guidelines. All qualitative studies of men’s experiences with body image, self-esteem, and masculinity whilst living with prostate cancer were included. A thematic meta-synthesis was conducted to identify emergent descriptive and analytical themes under the main study constructs.

**Results:**

Of 2188 articles identified, 68 were included. Eight descriptive themes were identified under two analytical themes: ‘Becoming a Prostate Cancer Patient’ and ‘Becoming a Prostate Cancer Survivor’. These described the distress caused by changes to body image, sexual functioning, sense of masculinity, and self-esteem, and the subsequent discourses men engaged with to cope with and manage their disease. A key element was increased flexibility in masculinity definitions, and finding other ways to re-affirm masculinity.

**Conclusions:**

Prostate cancer has an important effect on men’s health post-diagnosis, and we identified strong relationships between each construct evaluated. The role of hegemonic masculinity is important when considering men’s coping mechanisms and is also a key factor when addressing these constructs in counselling post-treatment.

**Implications for Cancer Survivors:**

This meta-synthesis provides key topics that uniquely affect prostate cancer survivors, enabling these patients to be effectively counselled, and have their concerns recognised by clinicians.

**Supplementary Information:**

The online version contains supplementary material available at 10.1007/s11764-021-01007-9.

## Background

Prostate cancer (PCa) is the second most frequent cancer in men worldwide [1], with an often long and indolent course. With 5-year survival rates at 83% in Europe, and improving, there is an increasing realisation that longer life does not always equate to living well [2]. Treatment side effects and the impact of the diagnosis itself may underlie the high prevalence of depressive and anxiety symptoms in men with PCa before, during, and after treatment [3]. Additionally, up to 60% of men experience mental distress during the course of their diagnosis or treatment [4], highlighting that the majority will experience effects on their mental wellbeing. The reasons for this are complex, with anxiety related to treatment decisions [5], distress related to PSA testing [6], and the impact of a cancer diagnosis [7] all likely contributing factors.

Although a large literature base exists evaluating the effect of PCa on defined mental health conditions such as depression and anxiety, less research has been done to identify other concepts that impact quality of life during and after treatment. Body image [8], self-esteem [9], and sense of masculinity [10, 11] are all impacted by a diagnosis of PCa, and are often impacted together: for example, body image and masculinity by androgen deprivation therapy (ADT) [12], or masculinity and self-esteem by erectile dysfunction [13]. Furthermore, it has been shown that men who adhere more strongly to hegemonic masculine scripts experience poorer mental health [14], suggesting these concepts may be both moderators as well as outcomes affected by PCa. This meta-synthesis aims to add to the existing literature on the effect of PCa on body image, masculinity, and self-esteem, and to try and establish whether there are possible links between these concepts.

When assessing the impact of interventions, tools are often used to measure effects on body image, self-esteem, and masculinity. However, it has been shown that current tools may not be suitable in cancer, and by extension PCa, patients as they may miss concepts unique to this group [15, 16], and furthermore, many remain unvalidated. Further qualitative research is required to set the foundation for the development and improvement of future quantitative detection tools evaluating these constructs. Therefore, this systematic review and meta-synthesis aims to (1) identify and explore the available qualitative literature evaluating body image, self-esteem, and masculinity constructs in men with PCa and (2) further understand the complex interrelationship between these three constructs. For the purpose of this review, the terms ‘men’ and ‘women’ have been used to refer to cisgender men and women.

## Methods

This systematic review was conducted according to PRISMA and ENTREQ reporting guidelines [17, 18], and was prospectively registered with PROSPERO (CRD42019157994) [19].

### Study eligibility criteria

Eligible qualitative studies were those that included adult PCa patients. This included papers that exclusively interviewed PCa patients, or studies that interviewed mixed cancer cohorts but stratified results by cancer so that responses only from the men with PCa were available. For the purpose of this meta-synthesis, body image was defined according to Hopwood’s research on body image in cancer patients, as an affective-cognitive-behavioural concept encompassing not only appraisal of physical appearance but also avoiding others, feeling less sexually attractive, and self-consciousness [20]. The concept of masculinity focussed on by this review was the social concept of gender, which is influenced by historical, social, and cultural factors [21], and reflects the differing view men may have about masculinity and how they embody this, or how it has been affected by a diagnosis of PCa.

Self-esteem was defined according to Rosenberg, with high self-esteem comprising considering oneself worthy and self-respect, and low self-esteem implying self-rejection, self-dissatisfaction, and self-contempt [22].

Studies required the availability of English data specific to men’s experiences with body image, self-esteem, and masculinity whilst living with PCa. We included studies which utilised either semi-structured interviews, open interviews, or focus groups, either in person or by telephone. All quantitative experimental or observational studies, conference abstracts with insufficient information, systematic reviews, and studies that did not use interviewing to collect data, e.g. questionnaires, were excluded. Studies that focussed on specific issues experienced by subgroups of men (e.g. exclusively homosexual men), and those evaluating treatment decisions or views on screening, were excluded as assessment of these was beyond the scope of this review.

### Information sources and search

PubMed, Embase, MEDLINE, and PsycINFO were searched for eligible studies from inception up until 6 May 2020. Three separate searches of keywords were carried out within each database. The 3 searches were (Prostate Cancer OR prostate neoplasm) AND (body image); (Prostate Cancer OR prostate neoplasm) AND (masculine OR masculinity); and (Prostate Cancer OR prostate neoplasm) AND (self-esteem). A reference review of relevant systematic reviews was also conducted, and the grey literature searched for via abstracts on EMBASE.

### Study selection

Once records were retrieved and duplicates removed, the remaining abstracts were screened for eligibility using EndNote, with full texts assessed by two reviewers independently (JB + OB). Any discrepancies were discussed until 100% agreement was reached.

### Data collection and synthesis

Initial data extraction consisted of elementary study data including date of publication, number of participants, country in which the study was conducted, treatment modality or stage of disease of the participants, research question, method of data collection, and method of analysis (Appendix Table 1). This extraction was carried out by two independent authors (JB + OB). For the qualitative analysis, both verbatim quotes from participants and analytical themes generated by study authors were coded. Where possible, quotes were coded before analytical themes so that primary data could be utilised as much as possible.

Qualitative data from the studies subsequently was analysed using thematic synthesis, first described by Thomas and Harden [23]. All eligible studies identified were exported to NVivo 12 software, and line by line descriptive codes were generated to describe common concepts. A list of initial codes was created, with subsequent data either added to these codes or used to generate new codes where necessary. These were then arranged into hierarchical descriptive themes. Descriptive themes were generated not only by an inductive process of rereading codes but also a deductive method of reviewing the research questions specified in the aims of the studies, and identifying previous studies which aimed to generate theory related to how health relates to the three constructs of interest [24]. This meta-synthesis used both inductive and deductive methods of coding, by utilising a template approach outlined by Crabtree and Miller [25] to order inductive codes under the deductive headings of ‘body image’, ‘masculinity’, and ‘self-esteem’, ensuring our analysis closely adhered to our research questions.

After data were coded and descriptive themes were formed, we generated analytical themes. This enabled the formation and discovery of additional concepts and hypotheses that may not have been identified in the primary data, and has previously been identified as a defining aspect of a qualitative meta-synthesis [26, 27]. Descriptive themes and their groupings under analytical themes were discussed between 2 independent reviewers (JB + OR) until full consensus was reached.

### Risk of bias assessment

The Critical Appraisal Skills Programme (CASP) tool for qualitative studies [28] was used to assess risk of bias in all studies included in this review. This evaluates the internal validity, the results, and the relevance to local populations for each study through ten items. To enable comparison between studies and to identify any studies with multiple obvious weaknesses, each study was assessed on the first nine items of the tool and given a percentage score for how many of the items were met. The tenth item of the CASP tool for qualitative studies (‘How valuable is the research?’) was not used due to its subjective nature. To enable comparison between studies and to identify any studies with multiple obvious weaknesses, each study was assessed on the first nine statements and given a percentage score for how many of the items were met with an arbitrary cutoff of 50% used to exclude studies from the review. The cutoff of 50% was pre-determined before critical appraisal took place, as recommended by the JBI manual for evidence synthesis [29].

## Results

### Study selection and characteristics

**Fig. 1:**
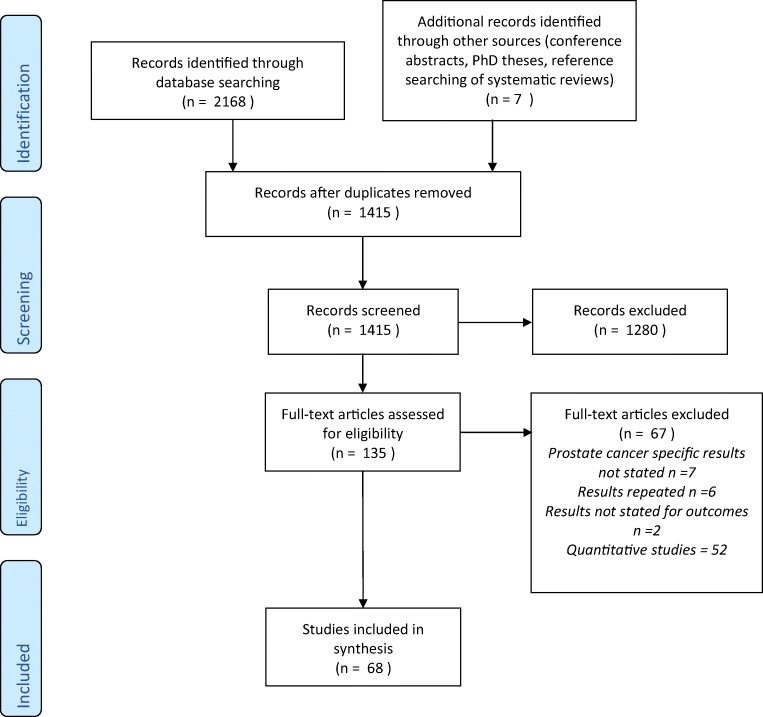
PRISMA flow diagram for included studies

A total of 2168 studies were identified from the initial search, with seven added through reference review and searching of grey literature (Fig. 1). After de-duplication and initial screening, 135 full-text articles were assessed for eligibility with 68 included in the final review.

Of the 68 studies, 36 addressed issues related to body image [11, 30–64], 68 masculinity [11, 30–96], and 50 self-esteem [11, 30–50, 52, 53, 55–59, 61, 62, 64, 66, 68–70, 72, 73, 78, 79, 81, 84, 86, 88–92, 94, 96], with most studies containing data relating to multiple themes. Studies took place in a total of 12 different countries, spanning five continents (Europe, North and South America, Oceania, and Asia). The total number of participants included in this review was 1443. Of these, 314 were interviewed in focus groups, 869 in semi-structured interviews, and 260 in open interviews. Exact cohorts varied, with some studies only accepting participants at a particular point post-diagnosis, receiving a particular treatment modality, or stratified by clinical stage of cancer. Additionally, other groups were often also interviewed in conjunction, including health professionals, consumer advisors, and patients’ partners [62, 64, 69, 89]. The overall age range of participants was 26–89 in studies reporting this (Appendix Table 1).

The CASP tool assessment demonstrated a range of quality of studies, ranging from meeting 67% (6/9 ‘Yes’) of the CASP qualitative criteria to 100% (median 89% IQR 78–89%). Questions 6 (‘Has the relationship between the researcher and participants been adequately considered’) and 7 (‘Have ethical issues been taken into consideration’) were those most often not met by 85% and 44% of studies, respectively. This was due to a lack of evidence for ethical review of the studies and no reflexive methods in the analysis of data to ensure the researcher and participant relationship was reflected upon. The results of the assessment are summarised in Table 1.

**Table 1:** Risk of bias assessed for all studies included, using the CASP qualitative tool. Numbers 1–9 refer to each question of the tool

Study	1	2	3	4	5	6	7	8	9	Percentage of CASP criteria met
Appleton et al. (2015) [11]	Y	Y	Y	Y	Y	Y	Y	Y	Y	100%
Araujo et al. (2013) [31]	Y	Y	Y	Y	Y	N	Y	Y	Y	89%
Araujo et al. (2019) [32]	Y	Y	Y	Y	Y	N	Y	Y	Y	89%
Araújo et al. (2019) [97]	Y	Y	Y	Y	Y	N	Y	Y	Y	89%
Arrington (2003) [65]	Y	Y	Y	Y	Y	N	Y	Y	Y	89%
Arrington (2003) [65]	Y	Y	Y	Y	Y	N	N	Y	Y	78%
Blanco (2006) [33]	Y	Y	Y	Y	Y	Y	Y	Y	Y	100%
Bokhour et al. (2001) [34]	Y	Y	Y	Y	Y	N	Y	Y	Y	89%
Bokhour et al. (2007) [67]	Y	Y	Y	Y	Y	Y	N	Y	Y	89%
Broom (2004) [35]	Y	Y	Y	Y	Y	N	N	Y	Y	78%
Broom (2005) [68]	Y	Y	Y	Y	Y	N	N	Y	Y	78%
Cayless et al. (2010) [36]	Y	Y	Y	Y	Y	N	N	Y	Y	78%
Cecil et al. (2010) [37]	Y	Y	Y	Y	Y	N	Y	Y	Y	89%
Chambers et al. (2018) [38]	Y	Y	Y	Y	Y	N	N	Y	Y	78%
Chambers et al. (2015) [69]	Y	Y	Y	Y	Y	N	Y	Y	Y	89%
Clark et al. (1997) [39]	Y	Y	Y	Y	Y	N	N	Y	Y	78%
Craike et al. (2011) [40]	Y	Y	Y	Y	Y	N	Y	Y	Y	89%
de Moraes Lopes et al. (2012) [41]	Y	Y	Y	Y	Y	N	N	Y	Y	78%
Dieperink et al. (2013) [42]	Y	Y	Y	Y	Y	N	Y	Y	Y	89%
Eilat-Tsanani et al. (2013) [43]	Y	Y	Y	Y	Y	N	N	Y	Y	78%
Ervik et al. (2012) [44]	Y	Y	Y	Y	Y	N	Y	Y	Y	89%
Evans et al. (2005) [45]	Y	Y	Y	N	Y	N	N	Y	Y	67%
Eziefula et al. (2013) [46]	Y	Y	Y	Y	Y	Y	N	Y	Y	89%
Farrington et al. (2019) [47]	Y	Y	Y	Y	Y	Y	Y	Y	Y	100%
Fergus et al. (2002) [48]	Y	Y	Y	Y	Y	N	N	Y	Y	78%
Gannon et al. (2010) [70]	Y	Y	Y	Y	Y	N	N	Y	Y	78%
Gentili et al. (2019) [49]	Y	Y	Y	Y	Y	N	Y	Y	Y	89%
Green (2019) [71]	Y	Y	Y	Y	Y	Y	N	Y	Y	89%
Hagen et al. (2007) [72]	Y	Y	Y	Y	Y	N	Y	Y	Y	89%
Hanly et al. (2014) [73]	Y	Y	Y	Y	Y	N	Y	Y	Y	89%
Hedestig et al. (2005) [50]	Y	Y	Y	Y	Y	N	Y	Y	Y	89%
Holmstrom et al. (2019) [51]	Y	Y	Y	Y	Y	N	N	Y	Y	78%
Jonsson et al. (2010) [74]	Y	Y	Y	Y	Y	N	N	Y	Y	78%
Kelly (2004) [75]	Y	Y	Y	N	Y	N	N	Y	Y	67%
Keogh et al. (2013) [76]	Y	Y	Y	Y	Y	N	N	Y	Y	78%
Kinnaird et al. (2020) [77]	Y	Y	Y	Y	Y	Y	Y	Y	Y	100%
Klaeson et al. (2012) [52]	Y	Y	Y	Y	Y	N	N	Y	Y	78%
Letts et al. (2010) [78]	Y	Y	Y	Y	Y	N	N	Y	Y	78%
Levy et al. (2015) [53]	Y	Y	Y	N	Y	N	N	Y	Y	67%
Maliski et al. (2008) [79]	Y	Y	Y	Y	Y	N	N	Y	Y	78%
Margariti et al. (2019) [54]	Y	Y	Y	Y	Y	N	Y	Y	Y	89%
Matheson et al. (2020) [80]	Y	Y	Y	Y	Y	N	Y	Y	Y	89%
McConkey et al. (2018) [55]	Y	Y	Y	Y	Y	Y	Y	Y	Y	100%
Medina-Perucha et al. (2017) [81]	Y	Y	Y	Y	Y	N	Y	Y	Y	89%
Mroz et al. (2010) [82]	Y	Y	Y	Y	Y	N	N	Y	Y	78%
Mroz et al. (2013) [83]	Y	Y	Y	Y	Y	N	N	Y	Y	78%
O’Brien et al. (2005) [84]	Y	Y	Y	Y	Y	N	Y	Y	Y	89%
O’Brien et al. (2007) [56]	Y	Y	Y	Y	Y	N	Y	Y	Y	89%
O’Shaughnessy et al. (2013)	Y	Y	Y	Y	Y	N	Y	Y	Y	89%
O’Shaughnessy et al. (2009) [57]	Y	Y	Y	Y	Y	N	Y	Y	Y	89%
Oliffe et al. (2007) [60]	Y	Y	Y	Y	Y	Y	Y	Y	Y	100%
Oliffe (2005) [58]	Y	Y	Y	Y	Y	N	Y	Y	Y	89%
Oliffe (2006) [59]	Y	Y	Y	Y	Y	N	Y	Y	Y	89%
Oliffe et al. (2009) [87]	Y	Y	Y	Y	Y	N	Y	Y	Y	89%
Oliffe (2009) [86]	Y	Y	Y	Y	Y	N	N	Y	Y	78%
Pietila et al. (2016) [88]	Y	Y	Y	Y	Y	N	N	N	Y	67%
Rivers et al. (2011) [89]	Y	Y	Y	Y	Y	N	Y	Y	Y	89%
Sartor et al. (2015) [61]	Y	Y	Y	Y	Y	N	Y	Y	Y	89%
Schantz Laursen (2017) [90]	Y	Y	Y	Y	Y	N	Y	Y	Y	89%
Stapleton et al. (2015) [91]	Y	Y	Y	Y	Y	N	N	Y	Y	78%
Ussher et al. (2017) [62]	Y	Y	Y	Y	Y	N	Y	Y	Y	89%
Ussher et al. (2017b) [63]	Y	Y	Y	Y	Y	N	N	Y	Y	78%
Wagland et al. (2019) [92]	Y	Y	Y	Y	Y	N	Y	Y	Y	89%
Walker et al. (2012) [64]	Y	Y	Y	Y	Y	N	N	Y	Y	78%
Wall et al. (2013) [93]	Y	Y	Y	N	Y	N	N	Y	Y	67%
Wennick et al. (2017) [94]	Y	Y	Y	Y	Y	N	Y	Y	Y	89%
Yu Ko et al. (2010) [95]	Y	Y	Y	Y	Y	Y	Y	Y	Y	100%
Zanchetta et al. (2007) [96]	Y	Y	Y	Y	Y	N	Y	Y	Y	89%

Index: *Y* ‘Yes’, *N* ‘No’

### Findings

**Fig. 2:**
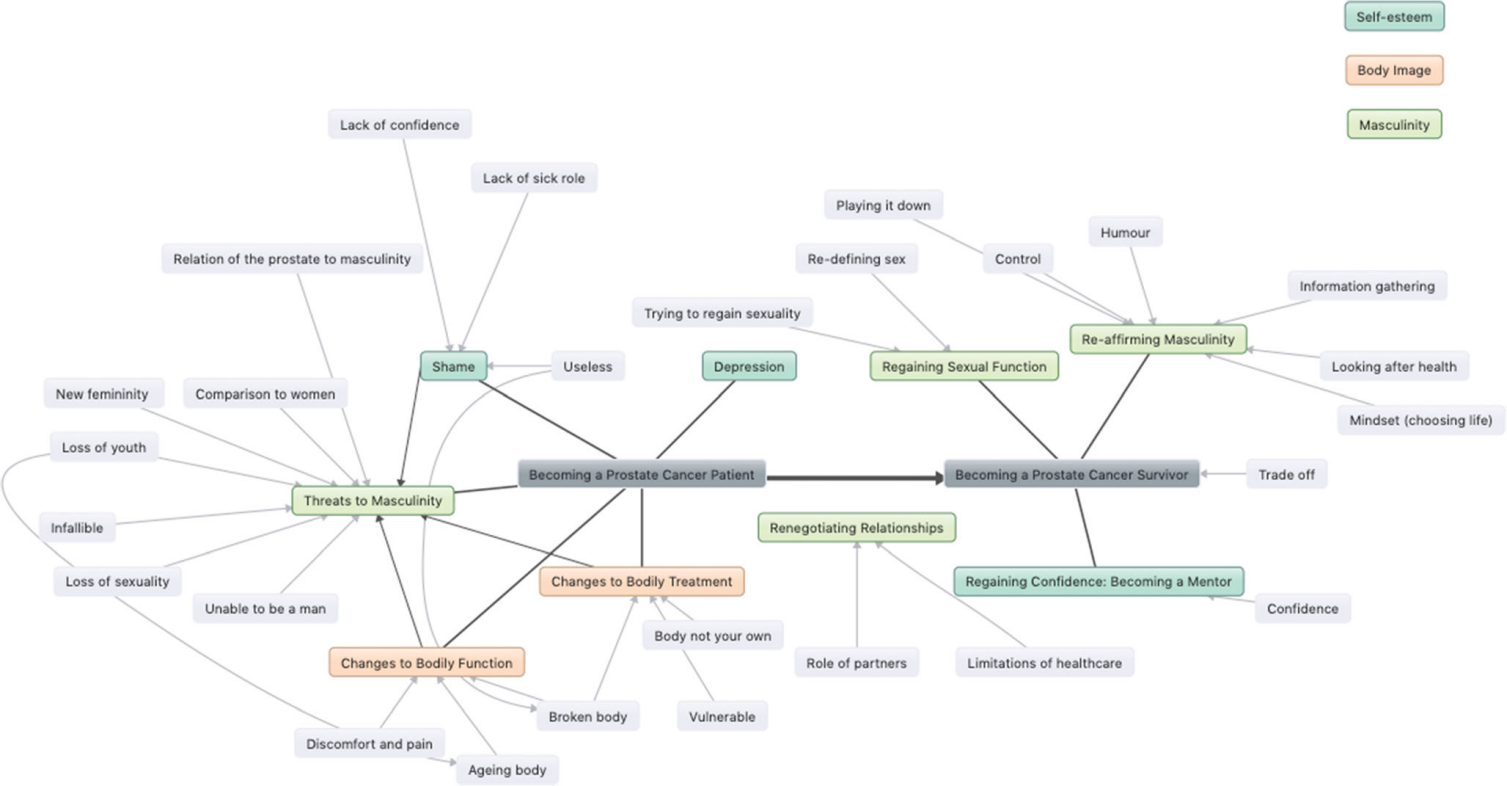
Mind map of second- and third-order themes identified, as well as individual codes in grey. Straight arrows show how codes contributed to the development of themes, and curved arrows show an interrelationship between concepts. Colour coding for second-order codes is shown top right

Thematic analysis identified a total of 31 codes (Table 2) and 8 descriptive themes under the headings of ‘masculinity’, ‘body image’, and ‘self-esteem’. These descriptive themes were subsequently arranged under analytical themes representing the PCa trajectory of ‘Becoming a PCa Patient’ and ‘Becoming a PCa Survivor’, reflecting the unique challenges and threats to emotional wellbeing encountered immediately after diagnosis and treatment, and the strategies men used to come to terms with these, often later into survivorship. Masculinity-related concepts were most frequently coded, with body image the least. Figure 2 demonstrates the relationships between codes, descriptive themes, and analytical themes identified.

**Table 2:** Summary of codes identified under each descriptive theme and frequencies

Code	Number of times coded
Body image	4
Ageing body	3
Body not your own	11
Broken body	50
Discomfort and pain	30
Masculinity	8
Comparison to women	41
Control	77
Humour	56
Infallible	11
Information gathering	71
Limitations of healthcare	71
Looking after health	23
Loss of sexuality	231
Loss of youth	71
Mindset (choosing life)	41
New femininity	26
Playing it down	89
Redefining masculinity	56
Redefining sex	41
Relation of the prostate to masculinity	14
Role of partners	133
Trying to regain sexuality	80
Unable to be a man	84
Self-esteem	11
Confidence	8
Lack of confidence	13
Lack of sick role	11
Shame	87
Useless	7
Vulnerable	29
Trade-off	77
Depression	20
Interference with daily life	32

### Becoming a prostate cancer patient

This first analytical theme generated from the review described the immediate changes men experienced to their masculinity, body image, and self-esteem, which were invariably negative. The themes under this were ‘threats to masculinity’, ‘changes to bodily function’, ‘changes to bodily treatment’, and ‘shame’. The second-order themes under this heading are demonstrated in Fig. 2.

This theme described men at the outset of their diagnosis, and was impacted the most by changes to sexual function. This negatively affected men’s sense of masculinity due to the preconceived ideals held that men should be able to function sexually in a natural, spontaneous, and unhindered way [30, 34, 48]. This change also affected their body image, as they realised their bodies now had reduced capabilities, further impacting men’s self-esteem. They lost confidence and withdrew from their former lives for fear of being found out as lesser men.

There were also new limitations men found in their lives—their bodies were weaker, and they had to limit their journeys outside due to fears of symptoms including incontinence or flushing [31, 37, 40, 41, 56]. Furthermore, this new limitation was not something they believed they deserved, with some describing themselves as infallible before their diagnosis, embodying the typical hegemonic male who does not seek healthcare or tell others about his problems [11, 35, 69, 81, 84, 85, 93, 98]. These men tried to hold onto these ideals even in the face of their illness, experiencing distress as they were unable to do so. They were faced with the choice to either hold onto their past lives, at the cost of their health, or accept their new masculinity.

This theme represents the conflict between retaining masculine ideals and accepting their new lives where this was no longer possible. This seemed to be held onto more at the beginning of their diagnosis, and those who strongly believed in concepts of hegemonic masculinity. Often men struggled to accommodate their illness, leaving them distressed, angry, and in some cases depressed.

### Becoming a prostate cancer survivor

The second analytical theme identified described the way men eventually began to accommodate and accept their new lives. Most importantly, this included how men began to change and reshape the way they saw masculinity, to enable them to continue feeling masculine despite the changes brought on by their illness. The themes under this were ‘regaining sexual function’, ‘renegotiating relationships’, ‘regaining sexual function’, ‘re-affirming masculinity’, and ‘regaining confidence: becoming a mentor’.

A theme often brought up by these men was the concept of a trade-off. It was most often discussed in terms of loss of sexual functioning, where men considered it acceptable to lose their masculinity in this way as a necessary measure to preserve their health [11, 35, 37, 38, 42–44, 46, 48, 50, 52, 56–59, 65, 71, 72, 77–79, 88, 94, 95]. Through this reasoning, men accepted not only their loss of sexual functioning but also more general aspects of their changed lives. This also meant that men were less likely to feel the need to try and regain their sexual functioning with aids, or find evidence to prove that they were sexually active in other ways, which were other coping mechanism for their functional loss. Conversely, some stated they preferred to risk their health in order to preserve their sexual function [35, 48, 54, 62, 65, 75], showing that although some men were able to engage in trade-off, others held onto masculine ideals so strongly that even threat to life could not make them abandon these.

This theme also embodied wider changes men made to their lives, and thought processes, to enable them to continue to live happily, and avoid the distress caused by their altered life. This involved the *re-affirming masculinity* theme, where men re-asserted their embodiment of masculine concepts in other areas of their lives, and *regaining confidence: becoming a mentor*, where men, instead of shying away from discussing their illness with others, began to mentor other diagnosed men, act as a representative for men with PCa, and advocate for increased research. Although this mentorship theme was at odds with the masculine ideals of stoicism and not seeking healthcare, it agreed with the ideals of power, strength, and leadership. Realigning themselves with some masculine ideals and abandoning others was a key part of *Becoming a PCa Survivor.* This theme required men flexible with their definitions of masculinity, but ultimately enabled men to be happier with their new lives, as they realised masculinity could be achieved in different ways.

### Masculinity

Masculinity concepts were present in all 68 papers [11, 30–96], and coded a total of 1311 times. Second-order masculinity themes tended to relate either to identifying threats to men’s constructions of their own masculinity, and the process of men realigning and redefining themselves so that they could continue to identify as men. These themes were *threats to masculinity*, *renegotiating relationships*, *regaining sexual function*, and *re-affirming masculinity*.

#### Threats to masculinity

A number of life changes presented threats to men’s masculinity. The most important of these was the inevitable change to sexual function and libido. Men described how procedures such as radical prostatectomy made them feel as if they had ‘lost a bit of [their] manhood’ [11], highlighting the belief that the prostate was an organ that differentiated men from women [32, 45]. Men compared radical prostatectomy to becoming ‘gelded’ [30, 48] or castrated [39], and were disturbed by the fact this procedure removed an organ they saw as vital [31, 35, 52, 97].

Changes to sexual functioning post-treatment was commented on often, being the most frequently coded code under the masculinity subheading. Men described themselves as ‘useless for sex’ [32], and were distressed by loss of libido as well as function [30, 31, 34, 37, 47, 48, 90]. Retaining sexual function was also so important to some men that they recounted choosing treatment depending on which offered the best potential for preservation of function [72], even if this reduced survival [35, 65, 75]. Multiple studies reported that men saw post-treatment erectile dysfunction as the most important factor they overcame [34, 54, 56, 89], with the shame of erectile dysfunction further compounded by incontinence disrupting their sexual relationships [55, 62, 80]. Decline in sexual function made men feel ‘not worthy’, less of, or unable to be a man compared to pre-treatment [30, 31, 33, 66, 77, 90].

For men receiving androgen deprivation therapy (ADT), side effects of treatment made them feel ‘like honorary women’ [47], or that they were ‘being turned into women’ [42, 46, 56]. Men described gaining ‘boobs’ [37, 59], demonstrating how men immediately saw gynaecomastia as a signifier of their feminine transition. Many also attributed emotional changes to ADT—crying more often [53, 80], becoming sentimental [59], and even wanting to bake more often [47].

The combination of these different factors made men feel that PCa had taken away their ability to be a man. Men described how PCa invalidated many of their ideals of who a man should be and what he should be able to do, representing a huge loss to their lives.

#### Renegotiating relationships

Partner relationships were frequently mentioned by men. Studies predominantly included heterosexual men, with only eight studies including gay or bisexual men [48, 55, 57, 62, 63, 73, 75, 80]. In men experiencing erectile dysfunction and loss of libido, the attitude of partners was a concern. Heterosexual men often posited their partners as uninterested in sex [59, 65], or admitted to not asking them how they felt about their changed sexual relationship [58, 78]. This enabled men to play down the effects of erectile dysfunction on their lives. Gay men faced more uncertainty in their relationships, describing the fact they could compare themselves with their partners, making them feel inadequate and embarrassed [48], and in some cases leading to the breakdown of relationships [62, 63].

Partners—almost always wives in these studies—played a key role in supporting men emotionally [37, 38, 44, 52]. They helped men both deal with the consequences of cancer [47], and encouraged them to seek out healthcare in the form of ‘nagging’ or ‘worrying’ [35, 37, 39, 42]. However, for single men, the effects of erectile dysfunction made them less likely to seek out new relationships [34, 38, 48, 80], end current ones [44], or to hide their diagnosis from romantic interests [65].

Relationships with healthcare professionals were often a new domain for men who were previously reluctant to seek out healthcare. Men found it difficult to discuss intimate issues like loss of sexual function with their doctors. This was due to these issues not being brought up by health professionals [62, 77, 78], and their own reluctance to reveal this loss of masculinity [55, 81]. Men described incompetence of surgeons as a cause for their erectile dysfunction [48, 67], as well as doctors who they believed had not conducted the appropriate tests [44], or failed to recognise their cancers earlier [38, 66]. Men also described inadequate information provided by physicians, particularly about sexual side effects, compounding their feelings that the healthcare received was somewhat to blame for their resulting loss of masculinity [11, 38, 42, 54, 61, 67, 72, 77, 78]. There was often also distress by their physician only being able to give limited advice on which treatment modality to choose, with this decision ultimately falling to themselves, and they therefore dwell on whether the ‘right’ decision was made [39, 60, 83].

The final relationship domain change was with other men. Men recounted that their lack of confidence due to physical changes, or simply reduced desire to be around others, meant that they withdrew from their social lives [36, 37, 50, 61, 66, 80]. There was often also no disclosure of their diagnosis to other men, instead keeping this to their partners [92].

#### Regaining sexual function

Use of sexual aids was mentioned by many groups [11, 33, 35, 41, 48, 55, 58, 63, 65]. Most men stopped using any sexual aids as they tended not to work [11, 65, 78, 89], or did not give men the spontaneous and natural erections that they desired, making them feel artificial and unnatural [30, 33, 34, 48, 57, 58, 70, 75, 90]. Furthermore, many described pain and discomfort associated with these devices [34, 58], making their use intolerable [63, 65]. However, for some, the use of sexual aids was acceptable, expressing increased confidence due to the fact they were now able to continue with their sexual lives [52, 62, 64, 73, 75].

For those not wanting to use sexual aids, but still desiring to maintain a ‘sex’ life (both for the benefit of their partners and to retain their sense of masculinity), sex was redefined as acts not requiring an erection or penetration. This was in contrast to the view many men had pre-diagnosis that penetrative sex was an important component of their masculinity [70]. Some began to include intimate acts such as hugging and kissing in their identity of being sexually active [38, 52, 58, 59, 62, 64, 65, 73, 90]. Others engaged in sexual activity not requiring penetration [33, 43, 48, 58, 70, 78]. Some men also maintained that being able to have sex without an erection was proof of being a superior lover, or resulted in them experiencing no loss of masculinity [65, 70, 94].

#### Re-affirming masculinity

Men engaged in a number of discourses to re-affirm their masculinity and normalise the changes caused by their PCa diagnosis. Control was a key feature of men re-affirming their masculinity. Men identified different ways in which they tried to re-exert control over their lives, making up for that which they had lost due to their diagnosis and treatment. Men described fear of their unknown futures [11, 32, 38, 44, 47, 80], requiring them to do something to try and regain power over their life trajectory. Many did this by gathering information about their disease from the Internet, book, and other men [31, 36, 38, 42, 43, 46, 50, 55, 56, 66–68, 70, 72, 74, 75, 96], as well as researching both current and future treatments [39, 44, 48, 53, 55, 60, 68, 75, 86]. Others looked for answers as to why they had the disease, making note of patterns of cancer in their families [36, 66]. PSA levels were a particular focus to some [36, 60], with worries that treatments may ‘mask’ their PSA score [47], which was seen as vital information to help them feel in control of their disease.

Another way men regained control was by ensuring families and spouses were protected from hardship, not only physically through funeral and financial arrangements but also emotionally, by sharing limited information about their condition and emotions [37, 53, 54, 92].

Taking a newfound interest in preserving their health also enabled men to feel as if they had more control over their disease, despite these behaviours being considered feminine by some [37]. Men described increased healthcare seeking [50, 79, 81, 84, 85], improved health behaviours, physical activity, diet [33, 37, 38, 50, 53, 76, 85, 86, 96], and paying increased attention to bodily changes [44, 58, 73, 84].

Humour has been previously identified as a feature of male discussion about cancer, where it is used to draw attention away from sensitive themes [99], and maintain masculine norms of stoicism and indifference to threats to health [100]. Humour was used directly by men in their interviews [42, 47, 62, 64, 72], and spoken about how they engaged with others [11, 35, 42, 48, 59, 67, 72, 87]. Joking about death and gallows humour was commonly mentioned, highlighting that playing down the emotional burden of their disease was another way to embody the masculine ideals these men subscribed to [38, 42, 67, 83]. Humour also presented a strategy to help men minimise the effects their PCa had on their sense of masculinity [72], particularly in situations such as focus groups and support groups, where they were encouraged to talk in detail about their problems in front of other men [60, 87].

Some men also justified changes to their masculinity by accepting them as consequences of getting older, particularly through normalisation of sexual and urinary symptoms [11, 33, 35, 42–44, 48, 52, 55, 57, 58, 61, 64, 70, 75, 77, 79, 96]. The increased vulnerability felt was also accepted as a consequence of older age [33, 37, 38, 56, 62], allowing them to reason that changes caused by PCa would have happened regardless. Conversely, some men emphasised the importance of believing in their own youth and ‘thinking young’ to delay the effects of both their cancer and general old age [63, 69], embodying the hegemonic concept that cancer was a disease men must fight against. For other men, confronting symptoms of PCa symbolised a premature ageing process, which distressed this group [41, 63, 71, 72, 79].

The importance of mindset was not only seen in relation to ‘thinking young’. Many appreciated a positive mindset was crucial to ensure their wellbeing. This embodied both thinking positively [40, 44, 46, 47, 50, 53, 69–73, 82, 88, 92, 96], as well as positioning cancer as a disease that should be ‘fought’ or ‘beaten’ [11, 59, 79], again allowing them to assume a dominant masculine role in the face of disease. Men also described previous experiences that gave them the ability to ‘handle’ their cancer diagnosis [11, 48, 69], enabling men to re-assert their strength and show that they had experienced worse than the challenges presented by PCa.

Another strategy employed by men to re-assert their masculinity was emphasising that their PCa had minimal effect on their lives, and by extension their masculinity. Men who lacked symptoms in particular were able to do this, using the lack of physical effects of disease as justification for why they should not be affected psychologically [11, 44, 65, 66, 69]. Men also described the concept of dying with PCa, rather than of it, as a reason for it not to affect them [11]. Others asserted it was simply something in life to be dealt with, despite negative feelings they may have [33, 36, 38, 41, 42, 48, 53, 70–72, 92]. This narrative was also used by those experiencing sexual dysfunction—by emphasising the unimportance of sex to them or their partners, they could downplay the effect this had on their lives [34, 37, 43, 65, 71, 75, 79]. Men identified that suppressing emotions in this way and downplaying the effect PCa had was a masculine trait [35, 38, 45, 78, 80, 81, 93].

The final strategy utilised was acceptance. That changes experienced in physical, sexual, and general health did not change the fact that they still identified as men. Men described that they were ‘still a man’ [11, 32, 33, 47, 48, 59, 79], despite the fact that loss of sexual function was seen as a key part of masculinity [34, 56].

### Body image

A total number of 107 body image-related codes were inputted, across 36 of the eligible studies [11, 30–64]. Men’s body image was affected by two main concepts: the changes in function they experienced due to their diagnosis and treatment, and the changes to how others treated their bodies. Therefore, the second-order codes under this heading were *changes to bodily function* and *changes to bodily treatment*.

#### Changes to bodily function

A wide range of changes to their bodies were described, with subsequent new limitations in their activities secondary to these. Physical changes such as fatigue [31, 37, 38, 49, 56, 61], urinary incontinence [31, 40, 41], and changes to appearance [37, 38, 49, 61] resulted in men perceiving their bodies as deficient [30, 37, 44, 45, 49, 50, 52, 62] and a source of shame [31, 41]. Even those experiencing limited functional changes felt that their body was now less than whole due to the removal of their prostate [32, 50, 52, 54, 62], or were distressed by extensive scarring [31, 40, 57]. Those undergoing ADT in particular described physical changes which meant their bodies were no longer their own [38, 59]. Body image was a concern not just for men who saw changes in their appearance but also those who experienced loss of function that they inherently associated with the capabilities of their bodies.

Physical discomfort became a part of men’s lives in a way they had not experienced before. Men described pain from treatments [11, 34, 36, 42, 43, 61] as well as the cancer itself [30, 36, 38, 42, 46, 51, 53, 61, 63, 64], creating the sense that men could no longer trust their bodies to function without some level of discomfort.

#### Changes to bodily treatment

A sense of loss of ownership over their bodies was common, as men began to engage with healthcare services more frequently. Letting others touch and handle their bodies for medical and surgical procedures created a new sense of vulnerability and shame [11, 30, 32, 35, 45, 60, 78]. Men also described seeing their bodies as old due to changes that were associated with premature ageing [33, 37, 38, 41, 42, 62, 72, 79]. Some men described in particular their experiences with catheters. This visible sign of illness, whilst accepted and accommodated for by some [11, 69], severely limited the lives of others due to loss of confidence and shame [30, 36, 41].

### Self-esteem

Self-esteem concepts were coded 186 times, across 50 eligible studies [11, 30–50, 52, 53, 55–59, 61, 62, 64, 66, 68–70, 72, 73, 78, 79, 81, 84, 86, 88–92, 94, 96]. Men described situations in which they now lacked confidence and factors that caused them to feel new shame. However, some found outlets, particularly through support groups and advocacy work, allowing them to regain lost confidence. The second-order codes under this heading were *shame* and *regaining confidence: becoming a mentor*. Experiencing depression was a distinct concept identified, although strongly linked to self-esteem and shame, which was mentioned by many as an effect of their diagnosis [44, 48, 50–52, 58, 61–64, 72, 73, 78, 80, 83, 94].

#### Shame

Shame was experienced in a variety of ways and settings. Their diagnosis itself was described as creating discomfort, with some feeling they lacked a sick role or recognition when they went into hospital [11], when other men did not believe they were unwell [37, 69], and particularly when others compared prostate to breast cancer [38]. This made men ashamed to admit their illness to others as they did not fit the traditional ‘sick’ model. Worthlessness due to being unable to work was also experienced, taking away the dominant masculine role they were used to [37, 41, 62].

Acute feelings of shame related to diagnosis was common, particularly from symptoms experienced, sometimes causing men to retreat from social situations or avoid leaving their house [30, 31, 38, 43, 59, 70, 80]. There was also a profound embarrassment caused by their inability to sexually perform in the same way as before [30, 33, 34, 41, 43, 45, 55, 62, 73]. This affected their relationships with their partners, and also caused a wider feeling of shame that meant men withdrew from all aspects of their social lives, feeling unable to talk about their experiences with others [11, 35, 38, 45, 52, 68, 70, 78, 79, 88, 90, 92, 94], including their own doctors [31, 73, 81]. This theme linked to ‘changes to bodily treatment’, as some diagnostic procedures contributed to men’s feelings of shame: rectal examinations were specifically identified by men as an embarrassing experience [35, 45, 81, 84]. Some men saw this as adjacent to homosexuality [52], creating a sense of shame associated with infringement of the masculine ideals they held.

A newfound vulnerability was also seen, due to physical changes [30–34, 38, 39, 52, 70], the loss of their social lives [30, 38, 48, 70], and the uncertainty surrounding the course of their illness [36, 44, 53, 58, 61, 66, 72]. Several also described an increased reliance on others [11, 39, 41], which some had previously looked down upon [35, 38, 66]. Although these men did not explicitly mention shame, their vulnerability was something they tried to hide, and was seen as a negative trait, again tying into the invalidation of their sense of masculinity.

#### Regaining confidence: becoming a mentor

Regaining confidence was described by some, mainly through PCa support groups. Five studies included instances where men either acted as spokespeople, drawing attention to PCa in their local communities [47, 53, 59, 72], or as a mentor for others within their support groups [86]. These men described their satisfaction and newfound positivity when they successfully convinced others to look after their health by undergoing investigations and attending screening: one man described that hearing others take his advice gave him ‘such a lift’ [47].

## Discussion

As survival rates improve, issues surrounding quality of life in PCa survivors are becoming increasingly pertinent. Strategies to improve these are imperative, and we provide an overview of three important interlinking aspects of this: masculinity, body image, and self-esteem. We identified 31 separate codes, used to generate eight second order, and the two main analytical themes for this review: *Becoming a PCa Patient* and *Becoming a PCa Survivor*. *Becoming a PCa Patient* described the changes to men’s body image, self-esteem, and masculinity that men saw as negative: factors such as loss of sexual functioning, increased vulnerability, and unwanted bodily changes. *Becoming a PCa Survivor* described the way men adapted to these changes, to either reframe them in a positive light, or accept them by redefining their ideas and expectations. The three outcomes of masculinity, self-esteem, and body image were connected by the fact that they were all affected by the loss of sexual function many men experienced, and the wide-ranging impact this had on men’s psychological wellbeing.

Previous reviews have examined the effect of interventions on body image and masculinity in PCa patients [101], evaluated only one of the three constructs [102, 103], or only studied a particular subset of patients [104, 105]. These reviews have previously also highlighted the threat to masculinity PCa can have and its importance as a barrier in seeking out healthcare. Similar to our findings, when evaluating coping and adjustment factors, Spendelow et al. identified ‘avoidance, minimisation, and withdrawal’ as well as ‘reframing masculinity’ as important mechanisms [103]. The relationship between bodily function and its effect on body image was also explored by our meta-synthesis. Fatigue and incontinence were considered to also reflect negatively on men’s bodies, as well as physical changes to appearance. The effect loss of function has on body image has been explored in other cancer cohorts: Rezaei et al.’s study of women with breast cancer found that muscle aches and reduced strength were associated with negative body image [106], whilst Fingeret et al.’s review of body image in cancer patients also recognised the impact of loss of function as well as changed appearance [107]. The lack of support by healthcare professionals after treatment has been reflected upon previously as well [108].

No previous review has evaluated more than 20 qualitative studies, as compared to the 68 included in this review. We are additionally the first to evaluate all three domains of body image, self-esteem, and masculinity in combination, thereby establishing a strong interlinking connection between them. The key theme that united these concepts was a loss of sexual function. This caused men to see their bodies as unable and inferior, negatively affecting both their confidence and body image, and also left men ashamed to talk to others about their experiences. Being able to function sexually was also seen as a key part of being a man and conferred masculinity; the loss of this was seen to affect almost every aspect of their identity.

The role of traditional hegemonic masculine ideals was central to our research, as proposed by Connell [109]. Connell defined this as a set of idealised practices including restricted emotional expression, power and success, stoicism, heterosexism, and misogyny [110]. Although many men may not challenge or conform to hegemonic masculinity, they display complicit masculinity, where they continue to benefit from men who demonstrate hegemonic masculinity. In the case of our meta-synthesis, men struggle when their diagnosis, symptoms, and treatment effects meant that they could no longer carry out these idealised practices, or challenged the ideals they had previously upheld as being masculine. Other theories of masculinity include the biological approach, which asserts that anatomy is what decides sex [111], and is reflected in some participants’ views that the physical removal of their prostate made them into less of a man. The social theory of gender assumes that masculinity, and by extension being a man, is a social construct taking into account historical, social, and cultural factors [21]. Meanwhile, social constructivist theories assert that masculinity is reinforced and embodied by the social interactions a man carries out [112].

Hegemonic masculine ideals were important in coping with a PCa diagnosis and in survivorship. These ideals men hold onto have implications for their adjustment and mental wellbeing post-diagnosis. Men who were inflexible in their ideals of masculinity were more distressed by changes experienced to their bodies and lives, and dwelled on losing part of themselves. Comparatively, those who were more flexible were able to re-affirm their sense of masculinity through other areas, or could justify their perceived loss of masculinity if it was the price for a longer life. These are important findings, providing areas to address when counselling patients, and should be discussed as a key strategy to improve quality of life during the survivorship period. Our findings are reinforced by previous quantitative research identifying that in older men diagnosed with cancer, high levels of distress were adjusted for when considering adherence to masculine ideals, with men strongly upholding these more likely to report depressive symptoms than their peers [113]. Similarly, previous reviews highlight reframing men’s concept of masculinity as a key theme in coping strategies used by men with PCa [103].

### Clinical implications

We have identified specific aspects of PCa that distress men during survivorship. By identifying these, clinicians can better understand the causes of adverse mental wellbeing in their patients, and also be aware of factors that discourage men from seeking healthcare. The *renegotiating relationships* theme of this meta-synthesis additionally highlights that some did not feel their doctors explained the consequences of treatment adequately. Considering the importance men placed on gathering information about their illness, this meta-synthesis demonstrates how imperative pre-treatment counselling and information about treatment options and their consequences is. This is particularly the case surrounding post-treatment sexual dysfunction, the common linking theme identified between our three investigated constructs.

The realities for men trying to use sexual aids to regain their erectile function was highlighted in a way which would not be possible quantitatively. These were often painful or difficult to use, with distress on failure and men still desiring the option to have ‘natural’ sex. This is contrasted with men who engaged in normalising discourses, or reframing of their concepts of masculinity and what sex meant to them. They reported less distress at their loss of sexual function and thereby a reduced impact on other constructs investigated. This highlights the importance of clinicians to support men coping with loss of sexual functioning psychologically as well as from a physical standpoint. There is a lack of psychosexual support for PCa survivors [108], despite psychological support being a key feature of adjustment to PCa [73], with demonstrated improvements in sexual satisfaction for survivors [114]. There is not only a need for improving support for patients but also how to best deliver this via further research as the current quality of evidence remains poor [115].

Our research has shown hegemonic masculinity may deter men away from screening and diagnostic procedures, as well as cause them to choose less effective treatment methods for the sake of preserving erectile function. We suggest this is incorporated into future public health messaging. The discourses men used to justify looking after their health should be emphasised, such as allowing them to be in control of their health, and utilise men already diagnosed with PCa in campaigns to normalise the diagnostic process.

A combination of these recommendations, along with the targeting of men’s traditional ideals of masculinity through therapy, should be incorporated into a cohesive local survivorship programme for men with PCa. The recognition of concepts that can both impair and improve men’s mental wellbeing after their diagnosis enables clinicians to recognise factors that may cause men distress and provide solutions to help improve wellbeing. Furthermore, more research is firstly required on the different experiences of men depending on which treatment modality they receive, and later on the effects of demographic factors such as age, location, and ethnicity. This would enable the creation of survivorship programmes tailored to specific patient groups to best suit men with PCa.

### Study limitations

This is the first meta-synthesis to evaluate the effects of body image, self-esteem, and masculinity in combination, and importantly the relationships between these, in men with PCa. We synthesised results from a wide range of populations to develop our analytical themes, with the hope that these can be used to help generate informed and specific tools to assess mental wellbeing outcomes and for targeted therapeutic areas in this cohort. Whilst this broader and larger sample is a strength, it also acts as a potential limitation by introducing significant heterogeneity into the findings. Many studies incorporated wide age ranges of participants, or did not state the age range in their paper. This may have introduced further heterogeneity to our results, as attitudes towards masculinity, body image, and self-esteem inevitably change between older and younger men. Furthermore, there was a paucity of studies that evaluated the effect of treatments such as active surveillance or chemotherapy on our chosen outcomes, limiting the generalisability of our results. Due to the large differences between different treatment pathways, and the ensuing effects on men who undergo them, further research, stratified by treatment modality, is essential to develop an all-encompassing model of how treatment for PCa affects patients.

Although findings are generalisable, they may not apply to specific PCa populations. Sexual dysfunction, for example, is likely to be less applicable to patients undergoing active surveillance, with distress at ADT symptoms not experienced by those undergoing surgery alone. Furthermore, many studies used convenience sampling, or specifically interviewed White, heterosexual men, with fewer studies focussing on Black Caribbean and African, or homosexual men. Therefore, ethnic minorities, homosexual, and bisexual men are underrepresented in this meta-synthesis. Many studies also did not state the ethnicity, age, or treatment modality undergone by their participants, creating ambiguity about the specific groups of PCa patients included in this meta-synthesis. Lastly, our meta-synthesis did not include transgender women who may also be diagnosed with PCa; to date, there have only been 10 documented cases of PCa in this group [116], with no qualitative literature identified. With the significant psychological impact of PCa highlighted by our meta-synthesis, we recommend further qualitative research in this specific patient group.

Although a rigorous search strategy was used, due to the wide variety in the sources of identified articles, there is also always a risk of studies being missed by this methodology. Our meta-synthesis did not search the CINAHL database, and did not include a search of MeSH terms, which may have missed relevant studies. Finally, our search strategy may not have identified all variations of the concepts of masculinity, body image, and self-esteem described by studies.

## Conclusions

We have summarised the available qualitative literature pertaining to men’s body image, self-esteem, and masculinity in patients with PCa. Key analytical themes of *Becoming a PCa Patient* and *Becoming a PCa Survivor* were identified. These described the adaptions and discourses men engaged with to avoid distress and adverse mental wellbeing outcomes from their diagnosis and treatment. The role of hegemonic masculinity in increasing risk of adverse mental wellbeing, due to the unique threat PCa presented to men’s constructions of masculinity, has also been demonstrated. Loss of sexual functioning, changes to the body, and feelings of shame and inadequacy are identified as important areas that require addressing during counselling and treatment of patients experiencing mental wellbeing problems. Future research should aim to build on these concepts, and identify the best therapeutic methods to address them once they arise.

## Supplementary information

ESM 1 (DOCX 58 kb)
