# Supplementary material for: Body image, self-esteem, and sense of masculinity in patients with prostate cancer: a qualitative meta-synthesis
Source: J Cancer Surviv. 2021 May 8;16(1):95–110. doi: 10.1007/s11764-021-01007-9 (PMC8881246; doi:10.1007/s11764-021-01007-9)
Supplement: Supplementary file 1 — (DOCX 58 kb) [file 11764_2021_1007_MOESM1_ESM.docx]

| First Author | Year of Publication | Country | Specific treatment group (if stated) | No of participants | Age range | Median Age | Mean Age | Data collection | Analysis | Research question? | Findings |
| --- | --- | --- | --- | --- | --- | --- | --- | --- | --- | --- | --- |
| Appleton et al | 2015 | UK | External beam radiation therapy (EBRT) | 27 | 57-76 |  |  | Semi-structured interviews | Grounded theory | "to explore how men receiving curative treatment managed their disease on a daily basis and what aided and hindered their ability to adjust to events and occurrences throughout the prostate cancer pathway" | Four areas were most important to the men: pathway to diagnosis, diagnosis itself, impact of PCa and treatment on daily life, and living with PCa. Men described how treatments such as hormone therapy made them feel less masculine. |
| Araujo et al | 2013 | Brazil | Either radical prostatectomy (RP) or chemotherapy | 20 |  |  |  | Semi-structured interviews | Content analysis | "To identify the social representations of men about prostate cancer and their masculinity to the disease, and analyse its implications for health. " | Concept analysis produced 3 main themes: ‘(re)knowing the prostate’, ‘this problem called prostate’, and ‘prostate cancer as a symbol of men’s masculinity’. |
| Araujo et al | 2019 | Brazil | RP | 17 | 61-81 |  |  | Semi-structured interviews | Thematic induction | “what is the experience of men regarding their body when developing PC?” | Prostate cancer presented a threat to men’s notions of hegemonic masculinity. Men underwent a transformation from healthy to sick, which affected not only their bodies, but their relationships with others and their own social values. |
| Araújo et al | 2019 | Brazil | Same as above | Same as above | 61-81 |  |  | Semi-structured interviews | Narrative synthesis | "it was sought to interpret the meanings attributed by men with prostate cancer to the experience regarding their bodies and masculinities during illness." | Men underwent transformations to both their bodies and their identities after they were diagnosed with PCa. They became subordinate to men with healthier bodies, and became marginalised in social relationships. |
| Arrington | 2003 | USA |  | 16 |  |  |  | Semi-structured interviews | Thematic analysis of narrative elements | RQ1: Do prostate cancer survivors' stories reveal changes in the storyteller's sexual identity or practice? What changes occur?; RQ2: How do themes found in prostate cancer stories relate to previous theories about illness narratives? Which do they confirm? Which themes suggest new directions in narrative theory? | Main events in the narrative of PCa were choice of treatment, and post-treatment changes in survivors’ sexual lives, either described as the end of their sexual life or the beginning of a modified one. Men with erectile dysfunction described the value of their health as more important than their desire for sex. |
| Arrington | 2003 | USA | Radiotherapy | 16 | 66-81 |  |  | Semi-structured interviews | Thematic analysis of narrative elements | RQ1: What common themes arise in the illness experiences of prostate cancer survivors? | Important events included diagnosis, information searching, making decisions about treatment, and post-treatment changes. Survivors believed the ‘cancer’ label was stigmatising and permanent, and described how they changed from uncertain pre-diagnosis, to becoming information seekers post-diagnosis. |
| Blanco | 2006 | UK | RP | 8 | 58-70 |  |  | Semi-structured interviews | Discourse analysis | "What are men's constructions of masculinity following radical prostatectomy?" | Men used 3 discourses to refer to their views of masculinity. Before diagnosis, they viewed themselves as mentally resilient, with an indestructible body that functioned naturally. They were unable to sustain this view once they began treatment, and instead justified their new vulnerability by accepting that this was inevitable with their illness and getting older. Men began to expand their definition of masculinity so that they could continue to define themselves as masculine. |
| Bokhour et al | 2001 | USA |  | 48 |  |  |  | Focus groups | Grounded theory | "to explore men's experiences and quality-of-life concerns associated with prostate cancer and its treatment" | Four domains of quality of life relating to sexuality were identified: the qualities of sexual intimacy, everyday interactions with women, sexual imagination and fantasy, and men’s perceptions of their masculinity. Erectile dysfunction affected both men’s relationships with others, but also their own self-image. |
| Bokhour et al | 2007 | USA |  | 2 |  |  |  | Semi-structured interviews | Sociolinguistic narrative approach | "This paper seeks to understand how men linguistically accomplish a narrative reconstruction of their lives in the discursive context of research interviews as they address the questions of (i) who am I?; (ii) how has prostate cancer impacted my life trajectory?; and (iii) how do I see myself in the face of fundamental changes in my functioning as a man?" | Men described PCa as a challenge to their personal, social and professional identities. Men saw themselves as breadwinners, sexually potent, and aggressors, and this identity was challenged by diagnosis and treatment for PCa. |
| Broom | 2004 | Australia |  | 33 |  |  |  | In-depth interviews | In depth exploratory approach (open coding and constant comparison) | exploring a group of men's experiences of being tested, investigated, and treated for prostate cancer, and their effects on men's sense of masculinity | Masculinity was a central concern when men considered what treatment to undergo for PCa, with men often prioritising this over more effective treatment when making decisions. Treatment and diagnosis of PCa presented a threat to men’s gender identities, and men were reluctant to talk about illness or weakness due to these being at odds with their idealised version of masculinity. |
| Broom | 2005 | Australia |  | Same as above |  |  |  | In-depth interviews | In depth exploratory approach (open coding and constant comparison) | The impact of internet use on the disease experience with prostate cancer, and the doctor-patient relationship | Men who accessed support and information online made them feel they could take control over the disease process, and removed some of the shame associated with face-to-face interactions. |
| Cayless et al | 2010 | UK |  | 10 |  |  |  | 3 Semi-structured interviews at diagnosis, treatment, and follow up | Grounded theory | "to provide an explanatory analysis of the liminal, ambiguous and chaotic experiences of men in this study, to outline the connexions with the concept of biographical disruption, and to identify the implications for supportive care." | Men diagnosed with PCa had to cope with ambiguity and uncertainty. Sexual dysfunction presented a threat both to the physical way men viewed their bodies as well as how they saw their own masculinity. Incontinence also presented a threat to men’s pre-diagnosis identity. |
| Cecil et al | 2010 | UK | No longer being actively treated | 8 | 36-70 |  |  | Semi-structured interviews | Content analysis | The impact of prostate cancer on representations of masculinity | Economic problems were of major concern to the men, as well as their changing relationships with their friends and family. Changes to their body due to treatment also negatively impacted on their body image. Men were able to justify their loss of libido for the preservation of their health. |
| Chambers et al | 2018 | Australia |  | 28 |  |  |  | Semi-structured telephone interviews | Thematic analysis | "To explore men’s lived experience of advanced prostate cancer (PCa) and preferences for support." | 2 organising themes were identified: lived experience, and supportive care. Lived experience encompassed regret about late diagnosis and treatment decisions,  being discounted in the health system, fear/uncertainty about the future, acceptance of their situation, masculinity and treatment effects, while supportive care had themes of communication, care coordination, accessible care, shared experience/peer support and involvement of their partner/family. |
| Chambers et al | 2015 | Australia |  | 15 |  |  |  | Semi-structured telephone interviews | Interpretive phenomenological analysis | "how masculinity, in the context of a prostate cancer diagnosis, might intersect with the life trajectory and life course; and given this proposed intersection how men with prostate cancer themselves define young as it applies to this specific illness experience." | Men defined young in relation to their diagnosis of PCa rather than in relation to chronological age. Men also defined young in relation to untimeliness of their diagnosis, their life course, and notions of masculine identity. Men considered PCa to be an “old man’s disease”. |
| Clark et al | 1997 | USA |  | 15 |  |  |  | Focus groups | Grounded theory | To evaluate the effect of symptoms on personal functioning and social relationships as perceived by patients who had undergone treatment | 3 major domains of quality of life were identified: self-perception, anxiety about the effects of treatment, and concern about the process of decision making and treatment. Indicators of PCa-associated quality of life included body image, sexual problems, masculinity, and cancer-related self-image. |
| Craike et al | 2011 | Australia | No longer being actively treated | 18 |  |  |  | Semi-structured interviews | Constant comparison | "to gain an in-depth understanding of the factors that influence participation in physical activity." | Barriers to activity tended not to be related to the physical effects of treatment. Instead, this was affected by factors such as lack of confidence, co-morbidities, and older age. |
| de Moraes Lopes et al | 2012 | Brazil | RP | 10 | 48-74 |  |  | Semi-structured interviews | Content Qualitative Analysis Technique | "to explore the psychosocial meaning and repercussions on lifestyle associated with erectile dysfunction and urinary incontinence (UI) in men following radical prostatectomy" | 3 themes identified: sexuality called into question, a body without governance, and experiencing loss. Men struggled both physically and mentally with sexual dysfunction, and the threat this posed to their sense of masculinity amplified feelings of powerlessness and psychological distress. |
| Dieperink et al | 2013 | Denmark |  | 13 |  |  |  | Focus groups | Phenomenological approach | RQ1: To explore participants' experiences of treatment with radiotherapy and ADT while participating in rehabilitation group; RQ2: Explore participants' thoughts and approach to spousal involvement in the rehabilitation process | ADT was seen to influence both sense of masculinity and identity. Men used humour to negotiated difficult topics, and the supportive role of spouses was emphasised. |
| Eilat-Tsanani et al | 2013 | Israel | RP | 22 | 60-81 |  | 73 | Semi-structured interviews | Creation of coding categories | "To describe the outcomes of radical prostatectomy as perceived by the patients, and their ways of coping with them." | Post-surgical sexual dysfunction caused feelings of shame, guilt, and decreased men’s self-esteem. However, men still saw surgery as a life-saving, necessary treatment. |
| Ervik et al | 2012 | Norway | Androgen deprivation therapy (ADT) | 10 | 56-83 |  |  | Semi-structured interviews | Phenomenological approach | "to illuminate how prostate cancer patients receiving endocrine therapy experience their bodily alterations through the course of the illness" | 5 themes identified: something is wrong, when the body becomes troublesome, to be well or to be ill, dealing with alterations, and talking about cancer and the intimate details. At first men were shocked by their diagnosis and worried by the physical changes their bodies went through. Later this gave way to worries about side effects which affected their everyday lives. |
| Evans et al | 2005 | Canada |  | 3 |  |  |  | Focus groups | Thematic analysis | "to explore the cultured and gendered dimensions of African Nova Scotian men and women’s experiences of breast and prostate cancer." | Men placed emphasis on sexuality and sexual performance as a measure of their masculinity. This made them reticent to attend screening appointments due to its violation of heterosexual norms, and made them see treatment for PCa as emasculating. Racism was seen to threaten masculinity, and caused men to receive a reduced standard of care compared to white men. |
| Eziefula et al | 2013 | UK | ADT | 19 | 45-84 |  | 68.8 | Semi-structured interviews | Framework approach | Qualitative exploration of flush-related cognitive appraisals and behavioural reactions reported by a sample of men with prostate cancer | 5 main cognitive appraisals identified: changes in oneself, impact on masculinity, embarrassment/social-evaluative concerns, perceived control, and acceptance/adjustment. Some men experienced shame and embarrassment due to fears about the perception of others, while others believed they invalidated traditional gender norms. |
| Farrington et al | 2019 | UK |  | 8 | 59-80 |  | 69 | Semi-structured interviews | Interpretive phenomenological analysis | "What is the lived experience of adjustment to prostate cancer?" | 4 themes generated: living with uncertainty/PSA test worry, renegotiating masculinity, the prostate champion, and humour. Adjustment to life with PCa involved dealing with changes to perceived masculinity, and engaging with concepts such as humour and becoming a prostate champion helped men to negotiate this. |
| Fergus et al | 2002 | Canada |  | 18 | 57-75 |  | 65 | Semi-structured interviews (4-5, 2 weeks apart) | Grounded theory | To explore the experiences of men living with sexual dysfunction as a result of treatment for prostate cancer | Core theme identified was preserving manhood. Within this were 5 major themes: enhancing the odds, disrupting a core performance, bearing an invisible stigma, effortful-mechanical sex, and working around the loss. |
| Gannon et al | 2010 | UK | RP | 7 |  |  |  | Semi-structured interviews | Foucauldian Discourse Analysis | To determine experiences and concerns following prostatectomy | 4 main discourses drawn upon: masculine identity and sexual activity, erectile dysfunction as a normative experience, mental resilience, and vulnerability. Penetrative sex was central to masculine identity. Men who were unable to achieve this normalised it as part of the ageing process. |
| Gentili et al | 2019 | UK | ADT | 22 | 43-85 |  | 67.9 | Semi-structured interviews (13 telephone; 9 in person) | Constant comparison | "the impact of ADT side‐effects on PCa patients' body image and sense of masculinity." | Men felt their bodies were feminised by treatment, which caused dissatisfaction with their appearance. Exercise was used to not only try and reverse the effects of ADT, but also to exert control over their bodies, and experience a sense of achievement. |
| Green | 2019 | UK |  | 29 | 53-83 |  |  | Open interviews | Thematic analysis | To explore men's experiences following treatment for prostate cancer through the lens of chronic illness | Men emphasised multiple masculine views, such as trying to show that they were in control of their illness, and had made the right decision by choosing treatment. Men who were part of PCa support groups aligned this with their ideals of hegemonic masculinity, by affirming that this required courage, survivorship and commitment to recovery. |
| Hagen et al | 2007 | Canada |  | 15 | 49-80 |  | 63.7 | Narrative interviewing | Phenomenological approach | "To explore the lived experience of men with prostate cancer" | Men described diagnosis and treatment for PCa as a journey, starting with shock at their initial diagnosis, progressing to information seeking and confronting various threats to their masculinity, and concluding with reflecting on the lessons they had learned. |
| Hanly et al | 2014 | Australia |  | 21 | 50-69 |  |  | Semi-structured interviews | Thematic analysis | "To explore factors influencing psychosexual adjustment, self-perception, and unmet information and support needs of prostate cancer patients and their partners" | 3 interconnected main themes identified which contributed to psychosexual adjustment: psychosexual impact, communication and support, and integration processes. Sexual and urinary dysfunction tainted men’s self-perception and damaged their personal relationships, while adequate communication and support from healthcare professionals and partners facilitated their adjustment to life with PCa. |
| Hedestig et al | 2005 | Sweden | RP | 10 | 61-69 |  |  | Narrative interviewing | Content analysis | "to illuminate the experience of living after radical prostatectomy for localized prostate cancer" | Men suffered from worry, anxiety and distress, and wished they could return to their pre-diagnosis lives. Incontinence and erectile dysfunction made men feel that they had lost their sex lives, changing their intimate relationships, and lowering their self-esteem. Men strived to gain control over their new lives. |
| Holmstrom et al | 2019 | USA |  | 19 | 51-78 | 67 |  | Semi-structured telephone interviews | Grounded theory; ISPOR guidelines for concept elicitation research | Aimed to develop a conceptual model to describe patient experience of living with metastatic castration-resistant prostate cancer | Most prominent symptoms for men were urinary frequency and urgency, fatigue, pain and stiffness, and sexual dysfunction. These interfered with daily activities, caused frustration, anxiety and sleep problems. Men with metastatic castration-resistant PCa also experienced enlarged breasts, muscle loss, inability to focus, interference with work, reduced body image perception, and lack of motivation. |
| Jonsson et al | 2010 | Sweden |  | 22 | 50-85 |  | 68 | Semi-structured interviews | Hermeneutical interpretation | "To provide information about if and how prostate cancer affects men's daily lives 2 years after diagnosis" | Men felt healthy but their lives were still impacted by their PCa diagnosis. The 3 themes identified were:  ‘age is claiming its due’, ‘living with uncertainty’ and ‘strengthening self-esteem’. The theme underlying these concepts was ‘balancing a changed life situation’. |
| Kelly | 2004 | UK |  | 14 | 52-77 |  |  | Ethnographic approach (periods of observation as well as interviews with men and healthcare professionals) | "The narrative and observational data were combined to produce broad data categories until sufficient data were obtained to produce a detailed account" | To explore the impact of prostate cancer on men's bodies and their everyday lives | Men assessed embodied risks (such as impotence) in highly personal ways, with some choosing to reject treatments based on their risk of impotence or incontinence. Despite support from health professionals, men still described a high level of uncertainty about treatment options. |
| Keogh et al | 2013 | NZ |  | 14 |  |  |  | Focus groups | Thematic induction | "to gain a richer understanding of older prostate cancer survivors’ perceptions regarding their quality of life and physical activity levels and benefits and risks of physical activity." | Primary themes identified were sexual health, ‘plumbing’ and non-urogenital side effects, return to and increased levels of physical activity post-diagnosis, physical health/function, psychological benefits of physical activity, as well as over-doing it and age-related risks of excessive physical activity |
| Kinnaird et al | 2020 | UK | EBRT | 8 |  |  | 74 | Semi-structured interviews | Thematic analysis | To explore men's perceptions of sexual dysfunction, including factors such as self-perception, relationships, and information and support needs | All men experienced sexual dysfunction following treatment. The main themes arising from analysis were: sexual issues not being a priority when choosing treatment, lack of information and support about sexual dysfunction, and the impact of sexual dysfunction on self-perception and relationships. |
| Klaeson et al | 2012 | Sweden |  | 10 |  | 65 |  | Phenomenological interviews | Phenomenological approach | To explore how middle aged (40-65) men diagnosed with prostate cancer at any stage experienced their sexuality from a lifeworld perspective | Main theme identified was ‘having the elixir of life stolen’. This had 4 constituents: ‘something that no longer exists’, ‘threat to manhood’, ‘intimacy’, and ‘staged manhood’. Men felt that the ability to be intimate was their life elixir, and that treatment for PCa had taken this away. Men felt their loss of sexuality threatened their identities, but were able to renegotiate their sexuality through their relationships with their female partners. |
| Letts et al | 2010 | Canada |  | 19 | 49-74 |  | 65 | Semi-structured interviews | Framework approach | (1) What changes in their sexual well-being (i.e., affection, sexual desire, sexual arousal, erections, orgasmic consistency, sexual satisfaction) do men with prostate cancer experience? (2) What are men’s experiences of each of these changes, including their emotional impact? (3) What are men’s perceptions of the impact of their prostate cancer-related changes in sexual functioning on their partner’s sexual well-being? (4) To what extent are men provided with the information they need on the potential impact of treatment on their sexual functioning and on dealing with changes in their sexual well-being? | Some but not all aspects of men’s sexual wellbeing were affected by a diagnosis of PCa. Furthermore, the specific aspects that were affected varied highly between individuals. Nearly all of the men described negative changes that were distressing to them in their erections, orgasmic consistency, and sexual satisfaction, and many believed their sex lives were now over due to erectile difficulties |
| Levy et al | 2015 | UK |  | 5 |  |  | 58.2 | Semi-structured interviews | Interpretive phenomenological analysis | To explore men with advanced prostate cancer's practices to promote and maintain emotional wellbeing | Two superordinate themes found: ‘living with imminent and uncertain death’, and ‘holding onto life’. Men had a reduced sense of the future, sense of isolation, and uncertainty, and these all affected their well-being. Men pursued well-being by managing their emotions, taking care of their families, and renegotiating their purpose. They also emphasised the importance of taking action and problem solving. |
| Maliski et al | 2008 | USA |  | 95 | 50-70 |  |  | Semi-structured interviews | Grounded theory | "To develop a descriptive model of processes used by low income African-American/Black and Latino men to maintain masculine identity with prostate cancer treatment-related symptoms" | Men’s masculine identities were constructed from their experiences in early life. These identities were then challenged by a PCa diagnosis, and then had to be renegotiated so men could maintain their identity as a man. |
| Margariti et al | 2019 | UK |  | 8 | 51-75 |  | 64.4 | Focus group | Thematic analysis | "to understand the experiences of African-Caribbean men with respect to their discharge to primary care following successful PCa treatment and the challenges associated with survivorship." | 3 themes identified: ‘discharge – misconceptions and uncertainties’, ‘survivorship – challenges and ways of coping’, and ‘black men and PCa – real and potential discrimination’. Men felt there was a lack of clarity about what discharge actually meant, and were concerned by the quality of follow-up care they received. The men reflected on stereotypes of Black male sexuality, how this was affected by erectile dysfunction, and how their experiences with erectile dysfunction caused them psychological distress. |
| Matheson et al | 2020 | UK |  | 28 | 46-87 |  | 65.9 | Semi-structured telephone interviews | Thematic analysis | "To explore the experiences of men with prostate cancer identified as having psychological distress and to identify factors influencing distress." | Men with psychological distress experienced a sense of loss in a variety of domains. This included self (identity, masculinity and self-confidence), physical functioning, connections with others, and control. Levels of psychological vulnerability varied between men, and was contributed to by maladaptive strategies such as emotional concealment, help-seeking avoidance, and withdrawal. |
| McConkey et al | 2018 | Ireland |  | 8 | 49-66 |  | 55.6 | Semi-structured interviews | Gorgi's descriptive phenomenological method | "to add to the emerging body of literature on gay men with prostate cancer, to broaden the research base, and to increase healthcare professional (HCP) knowledge and understanding of the issues faced by gay men with prostate cancer." | 3 key themes identified: experience of diagnosis, the experience of the healthcare service, and sources of support. Subthemes within these included: shock of diagnosis, the generalist nature of information provided, sexual side effects and incontinence, cancer support groups, and masculinity and gay identity. |
| Medina-Perucha et al | 2017 | UK |  | 20 | 57-83 |  | 69 | Semi-structured interviews | Thematic analysis | "to further explore the barriers for help-seeking in older men with individuals who have been diagnosed and have undertaken treatment for prostate cancer." | 3 themes identified, all related to negative attitudes towards help seeking: ‘male gender role’, ‘fear of the health condition and medical and treatment procedures’, and ‘embarrassment as a consequence of health procedures’. The male gender role prevented men from help-seeking, as men were reluctant to make emotional disclosures, and tried to portray themselves as strong and resilient. |
| Mroz et al | 2010 | Canada |  | 14 | 48-78 |  | 66 | Semi-structured interviews | Constant comparison | "to describe men’s perceptions of their diets and diet changes in response to their prostate cancer, and illuminate the reasons underpinning diet changes (or lack thereof) in their recovery and self-care" | Men followed 4 main patterns of eating post diagnosis: ‘eating as usual’, ‘intensifying efforts’, ‘adding-on’, and ‘overhauling diets’. 4 main domains informed diet change: perception of pre-PCa diet, diet and health understandings, orientation towards PCa, and the need for “doing something”. Dietary ideals were framed as masculine, important, action-orientated and autonomous, and alignment to masculine dietary ideals influenced whether men engaged in diet change. |
| Mroz et al | 2013 | Canada |  | 25 |  |  | 68 | Semi-structured interviews | Interpretive descriptive approach | To describe connections between masculinities and patients' perspectives of male patient-physician communication in the context of active surveillance | Most participants reported that communication from their physician about PCa was brief. Physicians were established in a position of authority and control, and patients were stoic in accepting active surveillance. Some participants were confused about their diagnosis and what active surveillance entailed, and desired collaborative decision making with their physicians. Men drew on masculine ideals such as stoicism, denying illness, and respecting expertise to inform how they negotiated their relationship with their physician. |
| O'Brien et al | 2005 | UK |  | 4 | 52-70 |  |  | Focus groups and semi-structured interviews | No named method specified: rereading of transcripts to identify themes | To investigate whether a mental illness (i.e., depression) presents different challenges to masculinity than those experienced in relation to a stereotypically male disease (i.e., coronary heart disease) and a gender-specific disease (i.e., prostate cancer) | There was widespread endorsement of the hegemonic ideal that men should be reluctant to seek help, especially amongst younger men. Help seeking was endorsed when it was perceived as a method to preserve or restore another, more valued, aspect of masculinity, such as sexual functioning, or maintaining a job. |
| O'Brien et al | 2007 | UK |  | Same as above | Same as above |  |  | Focus groups and semi-structured interviews | No named method specified: rereading of transcripts to identify themes | To determine the extent to which, and in what way, help-seeking behaviours are related to constructions of masculinity | Men felt that PCa removed essential aspects of their male identity, such as libido and sexual performance, and that these could not be recovered compared to social aspects of masculinity such as work. Men who were of working age more readily accepted their diagnosis, as they had more immediate concerns about their survival. While some engaged in practices to try and re-affirm their masculinity, most accepted they had lost part of their masculine identity forever. |
| O'Shaughnessy et al | 2009 | Australia | RP | 11 |  |  |  | Focus groups and semi-structured interviews | Content analysis | "to describe men’s long term recovery following prostatectomy for the purpose identifying the effects of unresolved post-surgical morbidity." | Fear, distress, loss, regret, anxiety, low self-esteem, depression, and changes in sexuality, masculinity, and relationships were described by the participants and their partners as adverse effects of both treatment and diagnosis. Wives were a key source of psychosocial support, and provided insight into the challenges men faced. |
| O'Shaughnessy et al | 2015 | Australia |  | 26 |  |  |  | Focus groups and semi-structured interviews | "Data broken down using tables, intuition, and comparison." | "to examine and understand the supportive care needs of men diagnosed and treated for prostate cancer at key identifiable periods of their cancer journey" | Incontinence and impotence were the main sources of emotional tension for men. These affected their social interactions and sense of self-worth. Men also expressed regret that they had not had access to information about the long term impacts of treatments. |
| Oliffe | 2005 | Australia | RP | 15 | 46-74 |  | 57 | Semi-structured interviews | Social constructionist gendered framework | To explore patients' experiences of impotence after prostatectomy | Men rationalised their loss of potency as a necessary process in order to live longer. Most men redefined masculine ideals of phallocentric sex, and focussed instead on activities such as shared interests and physical touch. |
| Oliffe | 2006 | Australia | ADT | 16 |  |  | 67.31 | Semi-structured interviews | Social constructionist gendered framework | To explore patients' experiences in the context of reduced testosterone that accompanies ADT | Participants reformulated hegemonic masculine ideals that they previously endorsed in response to changes to bodily functioning. They placed greater emphasis on aspects such as being a breadwinner, competitiveness and strength, and in some cases through their work campaigning for greater awareness of PCa. |
| Oliffe | 2009 | Australia |  | 3 |  |  |  | Semi-structured interviews and observation at prostate support groups | Ethnographic analysis | “How does masculinity inform and influence participants’ health and illness behaviours over time?” | Men took up health behaviours such as risk taking, reactive self-care, denial of illness, and the situating of females as primary health providers. Some of these behaviours were abandoned when men decided to promote their own and other men’s health. Men’s perceptions of masculinity changes over time as they both got older and were diagnosed with PCa, and some of their practices were informed by their upbringing and experiences when they were younger. |
| Oliffe et al | 2007 | Canada + Australia | | 52 | 47-89 |  | 64.92 | Secondary analysis of interview data from 2 older studies (Hinds, Vogel, & Clarke-Steffen, 1997; Thorne, 1994, 1998) | Inductive coding, with audit trail kept | 1. What patterns of communication do men with prostate cancer find helpful and unhelpful when communicating with male physicians about their illness? 2. How do prostate cancer patients preserve a masculine self when communicating with male physicians about their prostate cancer?" | Men acknowledged that compassion and expertise underpinned the patient-physician relationship, and enabled them to build trust. Reassurance and humour were identified as effective communication strategies. Men carried out a large amount of self-directed research about PCa to try and understand their disease and treatment options. |
| Oliffe et al | 2009 | Canada |  | 54 |  |  | 71.27 | Semi-structured interviews and observation at prostate support groups | Ethnographic analysis | to examine the connections between humour, health, and masculinities. | 4 themes identified: disarming stoicism, marking the boundaries, rekindling and reformulating men’s sexuality, and when humour goes south. Humour was used to promote inclusiveness, mark the boundaries for providing and receiving help, and to develop masculine group norms. There were some instances where humour would cause discomfort to attendees, although it was generally well received. |
| Pietila et al | 2016 | Finland |  | 20 | 50-76 | 65 |  | Thematic interviews and focus groups | Analysis of discursive practices | To explore men's justifications for treatment choices for localised prostate cancer | Those who underwent radical treatments conceptualised their illness as a lethal cancer, while those under active surveillance saw their cancer as less threatening. In both groups, men believed they made the only right choice. Men attached less masculine traits, such as irrationality and lack of self-reliance, to men who chose differently to them. |
| Rivers et al | 2011 | USA |  | 12 | 51-70 |  | 59.75 | Semi-structured interviews | Combination of content analysis and constant comparison method | "to identify and describe the most salient psychosocial concerns related to sexual functioning among African-American prostate cancer survivors and their spouses" | For the men their primary concern was quality of life, while female spouses considered survival as the most important outcome of treatment. Men were dissatisfied with the sexual side effects of treatment, but female partners were less concerned by this. |
| Sartor et al | 2015 | USA |  | 25 | 47-85 |  | 62.2 | Open interviews | Thematic analysis | " to identify and evaluate potential concepts that may be lacking from current disease-specific measures in prostate cancer" | Patients reported key symptoms such as bone pain, decreased urinary and bowel functioning, genital and muscle atrophy, reduced stamina, reduced body image, and effects of emotional wellbeing. |
| Schantz Laursen | 2017 | Denmark |  | 4 | 55-68 |  | 61.5 | Semi-structured interviews | Kvale's theory of interpretation of meaning (REF?) | "to elucidate the effect of surgical treatment for prostate cancer on men’s sexuality." | 4 themes identified from analysis: lack of control, sense of self, intimate relations, and redefining sexuality. Men felt that sexual dysfunction negatively impacted on their sense of self, intimate relations, and sex lives. They no longer saw themselves as ‘real men’, but some men were able to reframe their sex lives to encompass wider intimate acts, and emerged with stronger relationships because of this. |
| Stapleton et al | 2015 | UK | Palliative care | 8 | 26-68 |  |  | Open interviews | Collaizzi's seven-stage framework (REF) | "to understand how men experience their advanced cancer in relation to their perceptions of masculinity." | Themes included thwarted ambition, changing expectations, protection and provision, stoicism and coping, images of illness versus masculinity, the importance of being a fighter, and loss. Men benefited by sharing their experiences with other men, and used interviews as an outlet for their despair and grief at a diagnosis of advanced cancer. |
| Ussher et al | 2017 | Australia |  | 124 |  |  | 64.3 | Online survey and semi-structured telephone interviews | Thematic analysis | "Which sexual changes following prostate cancer are of concern to GB men with PCa and male partners? What are the meanings and perceived consequences ascribed to such changes? | Erectle dysfunction was reported by the majority of participants, and caused them to feel excluded from the sexual community of gay and bisexual men. This was also associated with emotional distress, and feelings of sexual disqualification. Other sexual concerns included loss of libido, climacturia, loss of sensitivity or pain during anal sex, non-ejaculatory orgasms, and reduced penis size. A number of men were reconciled to these changes and successfully re-negotiated their sexuality. |
| Ussher et al | 2017b | Australia |  | 38 | 45-78 |  | 64.6 | Semi-structured interviews | Thematic decomposition | RQ1. How do gay and bisexual men construct aging in the context of sexual embodiment after prostate cancer? RQ2. What are the consequences of constructions of aging for the subject positions men adopt in relation to sexual subjectivity? | 3 subject positions identified: mastering youth, which involved maintaining an active sex life through medical intervention, paying for sex, or having sex with younger men; the lonely old recluse, where men positioned themselves as prematurely aged and withdrawn from their previous social lives; accepting embodied ageing which involved accepting changed sexual function and incorporating this into relationships, while finding pleasure in non-sexual activities. |
| Wagland et al | 2019 | UK |  | 14 | 55-85 |  | 65 | Semi-structured telephone interviews | Framework approach | "To explore adjustment strategies adopted by Black African (BA) and Black Caribbean (BC) men in the UK as a response to the impact of PCa diagnosis and treatment effects." | 6 overarching themes identified: strong reliance on faith beliefs, maintaining a positive front, work as a distraction, non-disclosure of diagnosis due to stigma and beliefs about masculinity, raising awareness locally, and seeking support from close community. There were links between faith beliefs, presenting a positive front, community support seeking and locally raising awareness. |
| Walker et al | 2012 | Canada | ADT | 18 | 47-83 |  |  | Open interviews | Grounded theory | To understand the struggles couples faced when trying to adapt sexually to the side effects of ADT | ADT resulted in profound changes to men’s sense of masculinity and sexuality. Men experienced symptoms including erectile dysfunction, loss of libido, genital atrophy and severe genital shrinkage, hot flashes, loss of muscle mass, fatigue, and bodily feminization. This resulted in many men stopping all sexual activity, although some men continued to have satisfying sex despite their treatment. |
| Wall et al | 2013 | Australia |  | 8 | 40-85 |  | 63.4 | Semi-structured interviews | Constant comparison | "to explore the experience of men diagnosed with localized PCa during their first postdiagnostic year" | Men felt shock initially, and then worked to hide their distress by attenuating their feelings and trying to minimise the severity of their diagnosis. They did this because they believed it was expected of them, and believed it helped to protect their strong and stoic self-image. |
| Wennick et al | 2017 | Sweden | RP | 19 | 49-65 |  | 60.7 | Semi-structured interviews | Thematic analysis | "to illuminate how men under 65 years of age experience their everyday Life one year or more after a radical prostatectomy for localised prostate cancer." | 3 categories of experience identified: paying a price for survival, feeling sidestepped, and living with death lurking around the corner. Side effects such as sexual dysfunction resulted in men feeling as if they had lost their manliness, and reduced their self-esteem. They also felt that they did not receive enough support for what they considered to be an embarrassing disease. |
| Yu Ko et al | 2010 | Canada | RP | 6 | 58-77 |  | 64.7 | Semi-structured interviews | Grounded theory | "To provide an account of patients’ perceptions and responses to living with penile length shortening after radical prostatectomy" | No significant changes to masculinity or overall perception of self-image were reported. |
| Zanchetta et al | 2007 | Canada |  | 9 | 64-80 |  |  | Semi-structured interviews, genograms and ecomaps | Manual content analysis | "1. What ideas regarding PC occur across subsamples of men from different ethno- cultural backgrounds and emerge from the men’s experience with the four phases of PC experience? 2. To what extent do similarities exist between these groups of ideas regarding PC? 3. How do these ideas delineate hints of a core, transcultural representation of PC?" | Participants’ mothers had a strong influence on their health behaviours. Men felt uncertain about their futures after their diagnosis, but as they learned more about PCa they became more optimistic about their outlook. They also spoke openly with other men about their diagnosis, and were able to disclose their fears and difficulties, particularly with healthcare professionals. |

Appendix table 1: Table of study characteristics. Where information was not stated in a paper (e.g., median age) the column has been left blank.
